# The HIV-1 Tat Protein Induces the Activation of CD8^+^ T Cells and Affects *In Vivo* the Magnitude and Kinetics of Antiviral Responses

**DOI:** 10.1371/journal.pone.0077746

**Published:** 2013-11-04

**Authors:** Francesco Nicoli, Valentina Finessi, Mariaconcetta Sicurella, Lara Rizzotto, Eleonora Gallerani, Federica Destro, Aurelio Cafaro, Peggy Marconi, Antonella Caputo, Barbara Ensoli, Riccardo Gavioli

**Affiliations:** 1 Department of Life Sciences and Biotechnology, University of Ferrara, Ferrara, Italy; 2 Department of Molecular Medicine, University of Padova, Padova, Italy; 3 Department of Biomedical Sciences, Azienda Ospedaliero Universitaria Sant'Anna, Ferrara, Italy; 4 National AIDS Center, Istituto Superiore di Sanità, Rome, Italy; University of South Carolina School of Medicine, United States of America

## Abstract

T cells are functionally compromised during HIV infection despite their increased activation and proliferation. Although T cell hyperactivation is one of the best predictive markers for disease progression, its causes are poorly understood. Anti-tat natural immunity as well as anti-tat antibodies induced by Tat immunization protect from progression to AIDS and reverse signs of immune activation in HIV-infected patients suggesting a role of Tat in T cell dysfunctionality. The Tat protein of HIV-1 is known to induce, *in vitro*, the activation of CD4^+^ T lymphocytes, but its role on CD8^+^ T cells and how these effects modulate, *in vivo*, the immune response to pathogens are not known. To characterize the role of Tat in T cell hyperactivation and dysfunction, we examined the effect of Tat on CD8^+^ T cell responses and antiviral immunity in different *ex vivo* and *in vivo* models of antigenic stimulation, including HSV infection. We demonstrate for the first time that the presence of Tat during priming of CD8^+^ T cells favors the activation of antigen-specific CTLs. Effector CD8^+^ T cells generated in the presence of Tat undergo an enhanced and prolonged expansion that turns to a partial dysfunctionality at the peak of the response, and worsens HSV acute infection. Moreover, Tat favors the development of effector memory CD8^+^ T cells and a transient loss of B cells, two hallmarks of the chronic immune activation observed in HIV-infected patients. Our data provide evidence that Tat affects CD8^+^ T cell responses to co-pathogens and suggest that Tat may contribute to the CD8^+^ T cell hyperactivation observed in HIV-infected individuals.

## Introduction

Since its isolation in 1983, the human immunodeficiency virus (HIV) is still one of the major plagues worldwide with about 34 million of infected individuals and 1.7 million of deaths per year [1]. After almost 30 years of research, our understanding of HIV pathogenesis has progressed immensely, and we now know that progression toward disease depends on multiple parameters, including immunological, virological, intrinsic, genetic, as well as environmental factors. Studies on viral fitness and vaccine-development indicate that several components of the virus, including the so-called “regulatory proteins”, may contribute to the impairment of immune cells observed in HIV-infected individuals. During the course of HIV infection CD4^+^ and CD8^+^ T cells are functionally compromised despite their increased activation and proliferation [2-4]. Hyperactivation of T cells is one of the best predictive markers for progression toward AIDS and, although the causes are not fully understood, the forces that lead to immune dysfunction may differ for CD4^+^ and CD8^+^ T cells [2].

Tat is a regulatory protein produced very early after the HIV infection, necessary for viral gene expression, cell-to-cell virus transmission and disease progression [5-8] and can be released extracellularly [9-12] by a leaderless secretory pathway, even during antiretroviral therapy [13]. Upon release, Tat binds heparan sulphate proteoglycans of the extracellular matrix and is detected in the tissues of infected individuals [9,14] where it can exert its effects in non-infected HIV-specific and -non specific T cells. Furthermore, by targeting immune cells expressing RGD-binding integrin receptors via its RGD-binding site, extracellular Tat induces integrin-mediated signals and efficiently enters cells [14-16], resulting in the activation and modulation of several cellular functions in CD4^+^ T lymphocytes [6,7,17-22] and professional APCs [15,16], suggesting that Tat may play an important role in the chronic immune activation present during the HIV infection. However, whether Tat can affect CD8^+^ T cell responses and the *in vivo* antiviral immunity is not known. DCs are professional APCs central to the priming of CTLs, and CD4^+^ T cells help in the generation and maintenance of effector and memory CD8^+^ T lymphocytes; thus, it is reasonable to think that the Tat-mediated effects on these cell types could also impact the CD8^+^ T cell response and, thus, the control of infections.

Naïve CD8^+^ T cells recognize antigens presented as MHC-I peptide complexes by professional APCs and proliferate to generate a large number of effector CD8^+^ T cells that participate to the elimination of the pathogen. After this phase, called “expansion”, effector T cells undergo a “contraction” phase, leaving a small population of memory T cells having the potential to generate secondary responses after re-exposure to the antigen [23]. Both primary and secondary responses are affected by events occurring during the initial exposure (priming) to the antigen. It is known that activation of naïve CD8^+^ T cells requires multiple signals: signal 1, antigen-specific delivered via TCR/MHC interaction, signal 2, delivered by costimulatory molecules (including IL-2), and signal 3, delivered by pro-inflammatory cytokines and chemokines [23]. In this study, we sought to determine the effects of Tat on the kinetics and magnitude of primary and memory CTL responses in different *ex vivo* and *in vivo* models of antigenic stimulation. The presence of Tat at the time of the priming activated CD8^+^ T cells, enhancing effectors expansion and prolonging IFNγ release. However, CTL overstimulation turned to a partial loss of functionality at the peak of the response and to an effector memory phenotype at later time points.

These data provide evidence that Tat affects CD8^+^ T cell responses to co-pathogens, which may contribute to the immune activation and impaired control of infections observed in HIV-1 infection. 

## Materials and Methods

### Ethics statement

Experiments with animals were conducted according to European and Institutional guidelines for housing and care of laboratory animals and performed under protocols approved by the Italian Ministry for Health. 

### Tat and Gag proteins

The HIV-1 Tat protein from the human immunodeficiency virus type 1 (HIV-1) IIIB isolate (BH10 clone) was produced in *E. coli* and purified by diethylaminoethyl (DEAE) and heparin sepharose chromatography by DIATHEVA, as previously described [24]. The purified Tat protein was fully biologically active, as determined by the rescue assay on HLM-1 cell line carrying a Tat-defective HIV provirus [9,12], by the induction of transcription in TZM-bl cells, which contain a luciferase reporter gene under the transcriptional control of the HIV LTR and are commonly used to assess HIV infectivity, as well as by Tat uptake by monocyte-derived dendritic cells (MDDCs) evaluated by intracellular staining for Tat in flow cytometry [16]. This assay constitutes the potency test for HIV-1 Tat protein, since it is highly specific for the reduced form of Tat (uptake does not occur with the oxidized form), and is strictly dose-dependent, allowing a precise determination of the content of active protein in the preparation. LPS presence was assessed and endotoxin concentration was below the detection limit (0.05 EU/µg), as determined by the Limulus Amoebocyte Lysate analysis (Pyrochrome, Associates of Cape Cod, Falmouth, MA).

The HIV-1SF162 Gag (502 aa) protein was obtained from Novartis. 

The Tat protein was resuspended in PBS containing 0.1% of BSA for *ex vivo* experiments, while both Tat and Gag proteins were diluted in saline buffer containing 1% sucrose and 1% human serum albumin for *in vivo* experiments.

### Viruses

The wild-type Herpes Simplex virus type 1 (HSV1), LV strain [25], and a replication-incompetent HSV1 virus, named S0ZgJGFP, were used in this study. 

The S0ZgJGFP is a replication-incompetent HSV1 (Kos strain) derived from the replication-deficient S0Z virus [26], generated by a previously described transgene insertion procedure [27]. The S0Z virus carries deletions in two genes, including the *ICP4* immediate early gene, whose expression is essential for virus replication, and the *UL41* locus, encoding for the virion host shutoff protein that causes destabilization and degradation of cellular mRNA, and contains the *lacZ* gene, under the control of the HSV1 ICP0 promoter, in the *UL41* locus [26]. The recombinant replication-defective S0ZgJGFP virus, containing an additional deletion in the *Us5* locus, encoding for the envelope glycoprotein J (gJ), which plays a key role in inhibition of apoptosis in infected cells, was generated by homologous recombination between the *Us5* sequences of the replication-defective S0Z virus and of plasmid pTZgJHE. Briefly, plasmid pTZgJHE containing the *EGFP* gene, under the control of the human cytomegalovirus (HCMV) promoter, inserted in the *Us5* locus between Us5 flanking sequences, was generated by cloning the 2036 bp SalI-HindIII fragment, derived from the HSV-1 genome (nucleotides 136308 to 138345) and containing the *Us5* locus (corresponding to HSV1 gJ promoter and coding sequence), into the pTZ18U plasmid (Life Technologies). The resulting plasmid, pTZgJSalIHindIII, was used to generate plasmid pTZgJHCMVpr, containing a deletion in *Us5* [between the TATA box and the gJ coding sequence (nucleotides 137626-137729)] and the HCMV, derived from plasmid pcDNA3.1(+)Hygro as an NruI/SphI fragment, inserted into the SphI (137626) and NruI (137729) sites of pTZgJSalIHindIII. To obtain plasmid pTZgJHE, the *EGFP* coding sequence, derived from NheI/XhoI digestion of plasmid pcDNA3.1Hyg(+)EGFP, was cloned into the multi-cloning sites of pTZgJHCMVpr. 

The generation and isolation of the S0ZgJGFP virus were finally carried out in 7b cells, which are Vero cells stably transfected with HSV1 ICP4 and ICP27 genes, using the standard calcium phosphate transfection procedure with 5 µg of S0Z viral DNA and 1 µg of linearized plasmid pTZgJHE, as previously described [27]. The virus was purified by three rounds of limiting dilutions each followed by Southern blot analysis in order to confirm the deletion and the presence of the transgene.

### Large scale virus stock purification

Wild-type HSV1 and S0ZgJHE viral stocks were prepared by infecting Vero and 7b cells (4 x 10^8^), respectively, with 0.05 multiplicity of infection (m.o.i.) of virus in 15 ml of medium for 1 hour at 37°C under mild agitation. The virus was then removed and cells cultured at 37°C until a 100% cytopathic effect was evident. The cells were then collected by centrifugation at 2,000 rpm for 15 minutes. Supernatants containing the released viruses were spun at 20,000 rpm in a JA20 rotor (Beckman) for 30 minutes to collect the viruses, whereas the cellular pellets were resuspended in 2 ml of medium, subjected to three cycles of freeze-thawing (-80°C/37°C) and to a single burst of sonication to release the viral particles. The viruses were further purified by density gradient centrifugation (Opti Prep; Life Technologies, Inc.) and resuspended in PBS without calcium and magnesium. Viral stocks were titered *ex vivo*, according to standard procedures [28], and stored at -80°C.

### Peptides

Peptides were synthesized by solid phase method and purified by HPLC to >98% purity (UF Peptides, University of Ferrara). HSV1 K^b^-restricted peptides SSIEFARL (SSI), derived from glycoprotein B (gB), and QTFDFGRL (QTF), derived from ribonucleotide reductase 1 (RR1), were used to evaluate anti-HSV1 T cell responses in C57BL/6 mice. Gag K^d^-restricted peptides (Table S1) were used to evaluate anti-Gag T cell responses Balb/c and were provided by the NIH AIDS Repository Reagents and References Program. Peptide stocks were prepared in DMSO at 10^−2^ M concentration, stored at -20 °C, and diluted in complete medium before use.

### *Ex vivo* studies: cells preparation and culture

Splenocytes were isolated from C57BL/6 female mice (Charles-River Laboratories) as previously described [29]. Splenocytes from naïve mice were co-cultured, in the presence or absence of Tat (1 µg/ml), with autologous splenocytes pre-treated with mitomycin (25µg/ml for 1 hour at 37°C and washed three times) and loaded with the SSI peptide (10^-5^M for 1 hour at 37°C), in RPMI 1640 containing 10% FBS, 1% L-glutamine (BioWhittaker), 1% penicillin/streptomycine (BioWhittaker), 1% nonessential aminoacids (Sigma), 1 mM sodium piruvate (Sigma) and 50 mM beta-mercaptoethanol (Gibco). One million splenocytes pre-treated with mitomycin were used every 1.5 million splenocytes. After 8 days of co-stimulation cells were tested in IFNγ Elispot against the SSI epitope.

Alternatively, splenocytes, isolated from C57BL/6 female mice infected with 1 x 10^4^ of wild-type HSV1 (strain LV) and sacrificed at day 8 post-infection, were tested in IFNγ Elispot (see below) against the SSI epitope in the presence or absence of Tat (1µg/ml). 

### Mice immunization and infection

BALB/c female mice (Charles River Laboratories) were immunized with 5 µg of HIV-1 Gag protein alone or in combination with 5 µg of Tat protein. Immunogens (100 µl) were given subcutaneously, without adjuvant, at a single site in the back at day 0, and mice were sacrificed at day 10. C57BL/6 female mice were pre-treated, one week before infection, with 2 mg/100 µl of Depo-Provera® (Depo-medroxy-progesterone acetate; Pharmacia & Upjohn) subcutaneously in the neck, to bring the mice at the same estrous stage and render them more susceptible to HSV1 infection. Mice were inoculated intravaginally with 1 x 10^4^ of wild-type HSV1 (strain LV [25]), corresponding to 0.01 LD50, or 1 x 10^8^ of replication-defective HSV1 (S0ZgJGFP). Before injection, the virus was thawed on ice, sonicated for 5 seconds, and stored on ice. Mice were anaesthetized with 5% isofluorane to allow scraping of the vagina with a pipe scraper (in order to remove the mucus that could trap the virus) and then inoculated with the purified virus (in 10 μl of total volume for each mouse) using a pipette-tip. Mice were injected, at the time of infection, with Tat protein (5µg) given subcutaneously (Tat-treated mice), or with only Tat-suspension buffer (control mice).

After infection, mice were observed daily to monitor the appearance of local and/or systemic clinical signs of infection including death. Disease severity was measured using the following scores: 0 (no signs of infection), 1 (appearance of ruffled hair), 2 (appearance of cold sores on and around the vagina), 3 (appearance of paralysis of the back limbs) and 4 (mouse death). Mice were sacrificed at different time points to analyze anti-HSV1 immune responses on fresh splenocyte cultures (individual mice) by means of IFNγ Elispot assays or dextramer staining. At sacrifice, mice were anesthetized intraperitoneally with 100 µl of isotonic solution containing 1 mg of Zoletil (Virbac) and 200 µg Rompun (Bayer) to collect blood and spleens. 

At day 20 and 70 post-infection (p.i.), the presence of HSV1-specific IgM and IgG in sera was evaluated by enzyme-linked immunoassays (ELISA). Blood samples were collected from retro-orbital plexus, incubated for 16 hours at 4°C, centrifuged for 10 minutes at 10000 rpm to obtain sera and stored at -80°C until analysis. Each group was composed of three/five animals. Each experiment was repeated three times.

### Elispot assay

IFNγ Elispot assays were carried out using the murine kits provided by Becton Dickinson, according to the manufacturer’s instructions. Briefly, nitrocellulose plates were coated with 5 μg/ml of anti-IFNγ mAb for 16 hours at 4°C. Plates were then washed with PBS and blocked with RPMI 1640 supplemented with 10% FBS for 2 hours at 37°C. Total splenocytes from individual mice (1-5 x 10^5^ cells) were added to duplicate wells and incubated with HSV1- or Gag-derived peptides (10^-6^ M) for 24 hours at 37°C. Controls were represented by cells incubated with 5 μg/ml of Concanavaline A (GE Healthcare) as positive control or with medium alone (negative control). Spots were quantified using an AELVIS 4-Plate Elispot Reader (TEMA ricerca S.r.l.). The number of spots counted in the peptide-treated cultures minus the number of spots counted in the untreated cultures was the specific response. Results are expressed as number of spot forming units (SFU)/10^6^ cells. Values at least 3-fold higher than the mean number of spots in the control wells (untreated cells) and ≥ 50 SFU/10^6^ cells were considered positive. 

### Flow Cytometry

All stainings were carried out in FACS buffer (PBS containing 1% FBS) for 30 minutes at 4°C. The following antibodies were utilized: CD8 FITC (Immunotools); CD8 PE, CD4 FITC, B220 FITC, CD62L PE (eBioscience); CD8 PerCP, CD4 PerCP, CD95 PeCy7 (BD Biosciences); PD1 FITC (Biologend).

For dextramer staining, spleen cells (1 × 10^6^) were incubated for 10 minutes at room temperature with PE-SSI dextramer (Immudex) and washed prior staining with surface antigen antibodies. 

Data were acquired on a BD FACScan or a FACS Canto II and analyzed using BD Cell Quest Pro or Diva software.

### Enzyme-linked immunosorbent assay (ELISA)

 Anti-HSV1 specific antibodies in sera (IgM, IgG, IgG1 and IgG2a titers) were measured on samples collected from individual mice using 96-well immunoplates (Nunc Max Sorp) previously coated with 100 ng/well of HSV1 viral lysate (Herpes Simplex Type 1 Purified Viral Lysate, Tebu-bio), resuspended in PBS containing 0.05% NaN3, for 16 hours at 4°C. Plates were washed five times with PBS (pH 7.0) containing 0.05% Tween 20 (Sigma) (washing buffer) using an automatic washer (BioRad Model 1575 ImmunoWash) and then blocked for 90 minutes at 37°C by the addition of 200 µl/well of PBS containing 0.5% milk and 0.05% NaN3. After extensive washes, 100 µl/well of appropriate dilutions of each serum were dispensed in duplicate wells and then incubated for 90 minutes at 37°C. Plates were washed again before the addition of 100 µl/well of HRP-conjugated goat anti-mouse IgG (Sigma), diluted 1:1000, or HRP-conjugated goat anti-mouse IgM (Sigma), diluted 1:7500, in PBS containing 0.05% Tween 20 and 1% BSA, and incubated at 37°C for 90 minutes. In each plate, two wells were incubated with PBS containing 0.5% milk and 0.05% NaN_3_ and the secondary antibodies (blank). Analysis of anti-HSV1 IgG isotype was determined using a goat anti-mouse antibody directed against IgG1 or IgG2a (Sigma), diluted 1:30,000 in PBS containing 0.05% Tween 20 and 1% BSA. After incubation, plates were washed five times and subsequently a solution of 2,2'-Azinobis [3-ethylbenzothiazoline-6-sulfonic acid]-diammonium salt (ABTS) substrate (Roche) was added. The absorbance values were measured after 50 minutes of incubation at 405 nm with an automatic plate reader (SUNRISE TECAN). The cut-off value was estimated as the mean OD of 3 negative control sera plus 0.05. Each OD value was subtracted of the blank and cut-off values to obtain a net OD value. IgG titers were calculated by intercept function using the Excel program. 

## Results

### The HIV-1 Tat protein favors the activation of naïve CD8^+^ T cells

Several studies have reported the capacity of the HIV-1 Tat protein to increase, *in vitro*, IL-2 production in both HIV-infected and uninfected CD4^+^ T cells exposed to different stimuli [6,17,30]. Conversely, the role of Tat on CD8^+^ T cells, which are profoundly altered during HIV infection, is still unknown. 

**Figure 1:**
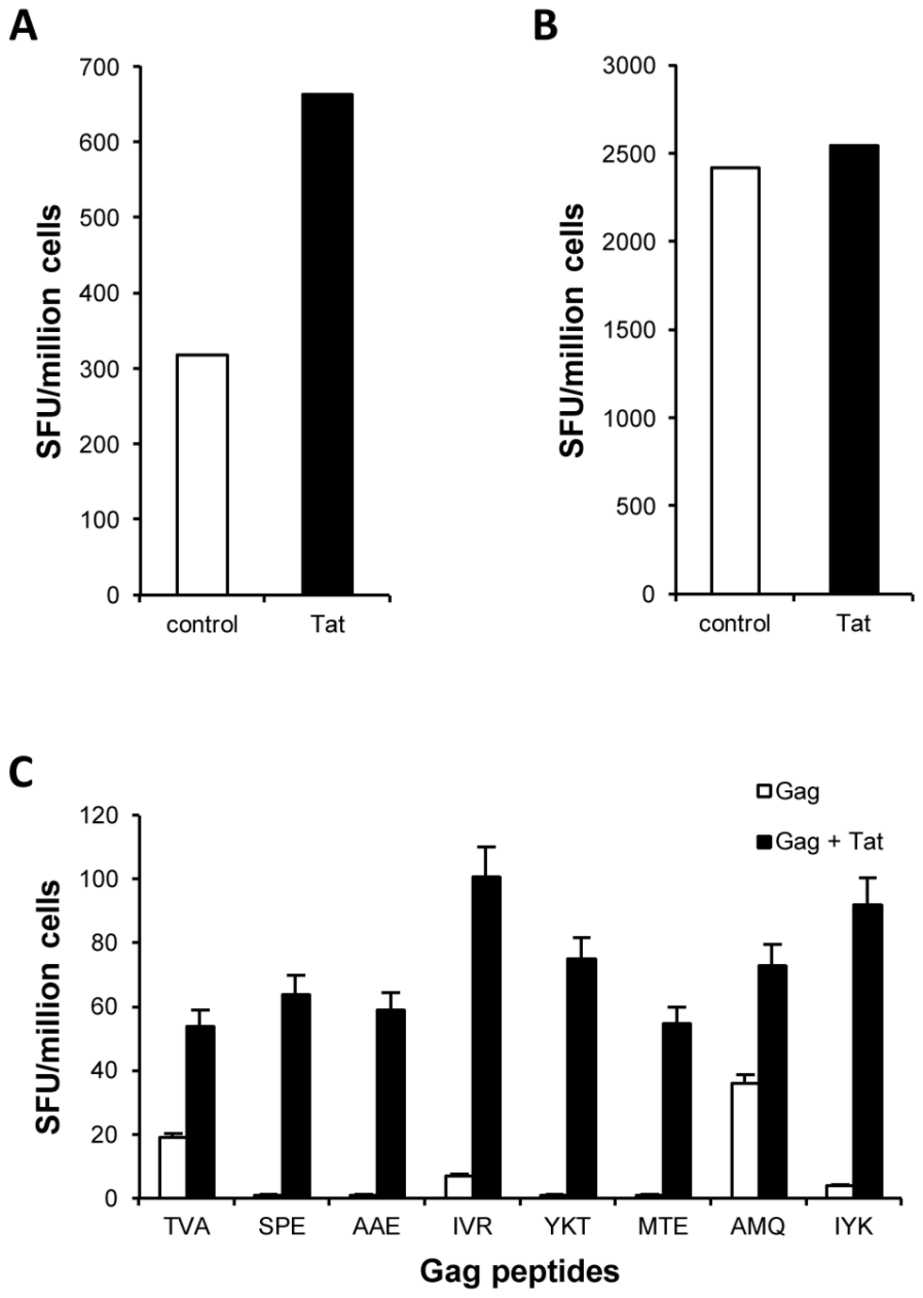
Tat-mediated CD8^+^ T cell activation. (**A**) Fresh splenocytes from C57BL/6 mice were co-cultured, in the presence or absence of 1µg/ml of Tat, with autologous splenocytes previously loaded with the SSI peptide epitope. After 8 days, cells were assayed in IFNγ Elispot. One representative experiment out of five is shown. (**B**) Splenocytes were purified from HSV1-infected C57BL/6 mice at day 8 post-infection and assayed in IFNγ Elispot against the SSI epitope in the presence or absence of 1 µg/ml of Tat. One representative experiment out of five is shown. (**C**) Balb/c mice (3 per groups) were injected with 5 µg of Gag alone or in combination with 5 µg of Tat. Ten days after vaccination mice were sacrificed and fresh splenocytes assayed in IFNγ Elispot against the indicated Gag-derived T cell peptide epitopes (see also Table S1). One representative experiment out of three is shown.

To determine whether Tat could affect CD8^+^ T cell responses, splenocytes from naïve C57BL/6 mice were cultured, in the presence or absence of the Tat protein, with autologous splenocytes previously loaded with the immunodominant K^b^-restricted CTL peptide epitope SSIEFARL (SSI) derived from the HSV1 glycoprotein B. After 8 days of stimulation, SSI-specific CTL responses were measured by IFNγ Elispot assay. As shown in Figure 1A, the presence of Tat induced higher numbers of IFNγ-secreting epitope-specific CD8^+^ T cells suggesting that Tat favors the *ex vivo* priming of naïve SSI-specific CD8^+^ T cells.

To discriminate whether Tat exerts its effects by enhancing the expansion of SSI-specific CD8^+^ T cells or potentiating the secretory capacity of effector CD8^+^ T cells, effector SSI-specific CD8^+^ T cells generated in absence of Tat, were assayed against the SSI peptide in the presence or absence of Tat. As shown in Figure 1B, the presence of Tat did not affect the release of IFNγ against the SSI epitope. These results suggest that Tat favors the priming and the expansion of CD8^+^ T cells but does not increase their effector function.

To confirm these *ex vivo* observations, the effect of Tat on the expansion of naïve CD8^+^ T cells was investigated using an *in vivo* model of protein vaccination based on the HIV-1 Gag protein that allows the characterization of T cell responses directed to several immunodominant and subdominant CD8^+^ T cell epitopes within the Gag antigen [29,31]. Specifically, Balb/c mice were immunized with the HIV-1 Gag protein alone or in combination with Tat and, ten days after immunization, fresh splenocytes from immunized mice were assayed by IFNγ Elispot to evaluate T cell responses directed against previously identified Gag-derived peptides [31] containing eight CD8 epitopes (Table S1). As shown in Figure 1C, immunization with Gag alone did not induce detectable Gag-specific T cell responses, whereas immunization with the Gag protein in the presence of Tat elicited significant Gag-specific responses directed against all tested CD8 epitopes. These data demonstrate that the Tat protein induces the expansion of antigen-specific CD8^+^ T cells. Tat-specific cellular responses measured by IFNγ fresh Elispot assays against overlapping 15-mer Tat peptides were absent in both groups of mice (data not shown), demonstrating that the enhancement of Gag-specific responses is unrelated to the induction of anti-Tat cellular response.

### The HIV-1 Tat protein increases the duration and decreases the intensity of antiviral CD8^+^ T cell responses

**Figure 2:**
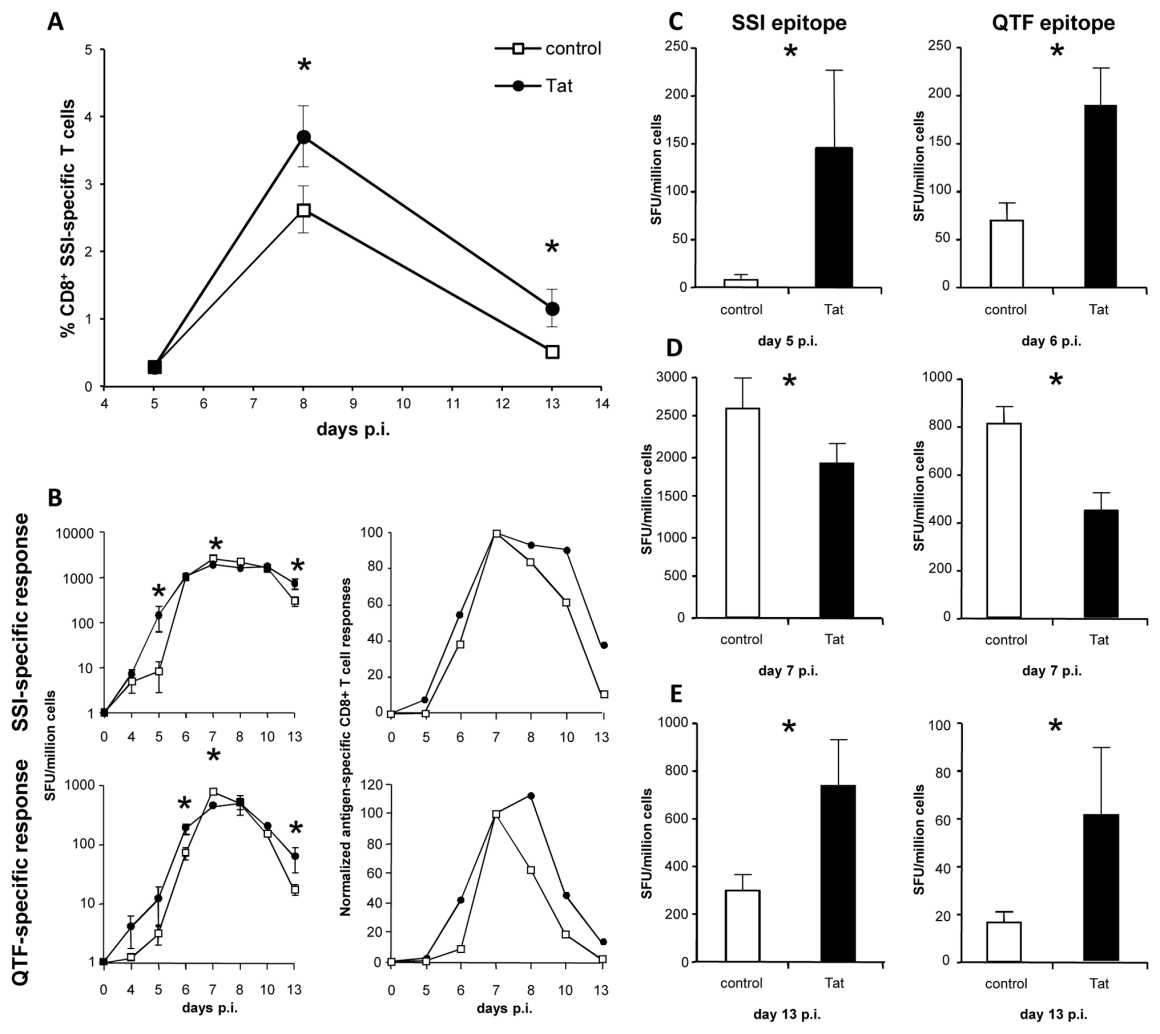
Tat modulates the kinetics and the magnitude of CTL responses. Splenocytes were purified from control and Tat-treated HSV1-infected C57/BL6 mice at days 4, 5, 6, 7, 8, 10 and 13 post-infection. (**A**) Percentage of SSI-specific CD8^+^ T cells detected by dextramer staining. Data are presented as mean ± SEM of 5 mice per group. One representative experiment out of three is shown. (**B**) Kinetics of SSI- and QTF-specific cellular responses detected by IFNγ Elispot on fresh splenocytes. Data are presented as mean ± SEM of 5 mice per group (left panel). Expansion and contraction were normalized, for each group, to values detected at day 7 (right panel). One representative experiment out of three is shown. (**C**) SSI- and QTF- specific IFNγ responses at days 5 and 6 post-infection. (**D**) SSI- and QTF- specific IFNγ responses at day 7 post-infection. (**E**) SSI- and QTF-specific IFNγ responses at day 13 post-infection. For statistical analysis two-tailed Mann Whitney test was used. *P<0.05.

The stimulatory effect of Tat on CD8^+^ T cells (Figure 1) and its capacity to increase antigen presentation, as reported in previous studies [15,16,20,29], suggest that Tat may play a role in the hyperactivation of CD8^+^ T cells observed during HIV infection as well as in the modulation of the immune response to co-infections. To understand how the immunomodulatory properties displayed by Tat on APCs and T lymphocytes affect, *in vivo*, the overall immune responses against a viral infection, C57BL/6 mice were infected intravaginally (i.v.) with wild type HSV1 (strain LV) with or without the Tat protein administered at the time of infection by the subcute route. At days 5, 8 and 13 post-infection (p.i.), the presence of HSV1-specific CD8^+^ T cells was evaluated on splenocytes by staining with MHC-peptide dextramers specific for the immunodominant SSI CTL epitope of glycoprotein B. As shown in Figure 2A, at day 5 p.i. low numbers of SSI-specific T cells were detected in both groups of mice. However, at day 8 p.i., when CD8^+^ T cell response reached the peak of expansion, and at day 13 p.i., corresponding to the contraction phase of the T cell response, the percentage of SSI-specific CD8^+^ T cells was significantly higher in mice treated with the Tat protein than that measured in the control group inoculated with HSV1 alone.

Since the presence of antigen-specific CD8^+^ T cells does not necessarily correlate to a functional cytotoxic phenotype [32,33], the activity of SSI-specific CD8^+^ T cells isolated from mice infected with HSV1, with and without Tat, was further investigated. Moreover, to better characterize the population of HSV1-specific CD8^+^ T cells, CTL responses against the subdominant QTFDFGRL (QTF) epitope of ribonucleotide reductase 1 (RR1) were also analyzed. To this purpose, fresh splenocytes purified from mice infected with HSV1, in the presence or absence of Tat, were tested at different time points p.i. by evaluating IFNγ release against the immunodominant SSI and the subdominant QTF epitopes. As shown in Figure 2B, the expansion phase of HSV1-specific CD8^+^ T cell responses against both epitopes was more robust in mice treated with Tat as compared to that observed in the control group. Indeed, significantly higher numbers of SSI- and QTF-specific CD8^+^ T cells secreting IFNγ were detected in the Tat-treated group at days 5 and 6 p.i., respectively (Figure 2C). 

Conversely, and despite the higher percentage of SSI-specific CD8^+^ T cells measured by dextramer staining (Figure 2A), at day 7 p.i., corresponding to the peak of the expansion phase of CTL responses, a significant lower number of SSI- and QTF-specific IFNγ-secreting cells was observed in mice treated with Tat compared to the control group (Figure 2B, D). To try to solve this apparent contradiction, the expression of CD62L and IL-7 receptor (CD127) was evaluated on SSI-specific effector T cells, since these markers define specific CD8^+^ subpopulations with different pattern of cytokine secretion [34]. In particular, during the expansion of CTL responses, the majority of antigen-specific CD8^+^ T cells are usually within the CD62L^-^CD127^-^ subset, consistent with the effector phenotype, while a small subset of T cells retains the expression of CD62L and CD127 molecules. The results of these analyses showed that the largest population of SSI-specific CTLs consisted in CD62L^-^CD127^-^ cells (Figure S1) in both groups, indicating that the lower number of IFNγ-secreting T cells in Tat-treated mice was not due to the unbalancement of effector cell subpopulations. It is likely that the robust expansion of HSV1-specific CD8^+^ T cells induced by Tat damps the cytokine release at the peak of the response, resembling the impaired functionality that follows hyperactivation of CD8^+^ T cells during chronic immune activation [35-37].

Finally, we analyzed SSI- and QTF-specific CTL responses during the contraction phase. As shown in Figure 2B, E, a significant higher number of SSI- and QTF-specific CD8^+^ T cells secreting IFNγ was detected in the Tat-treated group at day 13 p.i., demonstrating that Tat delays the contraction phase of the antiviral CTL response. Taken together, these data suggest that the presence of Tat at the time of priming results in primary CD8^+^ T cell responses that start earlier and last longer but have a lower intensity at the peak.

### Tat treatment does not contribute to the control of acute HSV1 infection

**Figure 3:**
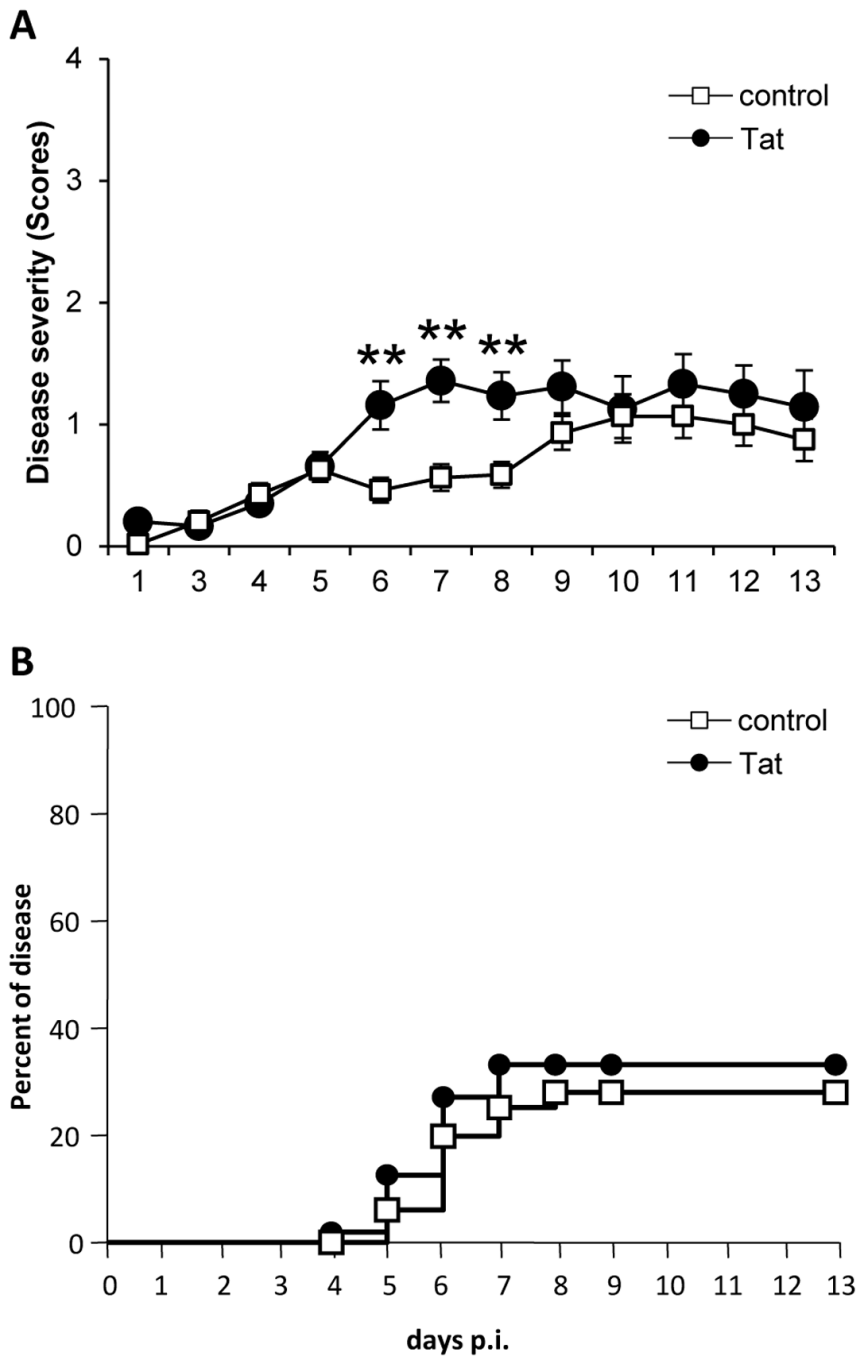
Tat does not contribute to the control of HSV1 acute infection. Control and Tat-treated HSV1-infected C57/BL6 mice were checked daily for the appearance of disease signs. (**A**) Mean of disease scores ± SEM of 20 mice per group is shown. For statistical analysis two-tailed Mann Whitney test was used. **P<0.01. (**B**) Probability of developing disease signs is shown for each group. Figure represents Kaplan-Meier estimation of the probability of clinical manifestations. For statistical analysis Log rank test was used. One representative experiment out of three is shown.

Several studies demonstrate that helper and cytotoxic T lymphocytes are functionally important in response to infection with HSV [38]. In particular, CTLs are critical in limiting the number and severity of herpes lesions, promoting recovery from primary and recurrent infections [39,40]. To determine whether the different CTL responses against HSV1 elicited in the presence or absence of Tat have an impact on the outcome of viral disease, HSV1-infected, control and Tat-treated, mice were monitored daily for the appearance of typical HSV1 clinical manifestations. Disease severity was measured using scores starting from no signs of infection (score 0), appearance of ruffled hair (score 1), appearance of cold sores on and around the vagina (score 2), appearance of paralysis of the back limbs (score 3) and mouse death (score 4). As shown in Figure 3A, the clinical manifestations were significantly more intense in Tat-treated mice between days 6 and 8 p.i., at the peak of the expansion of the cellular responses, when the animals infected in the presence of Tat showed a lower IFNγ release. However, mice from the two groups showed the same probability of developing signs of disease (Figure 3B). Taken together, these data suggest that the presence of Tat, although prolonging the CD8^+^ expansion phase, worsens acute disease manifestations and does not contribute to a better control of HSV1 infection. 

### Tat-mediated stimulatory effects involve only antigen-specific CD8^+^ T cells

**Figure 4:**
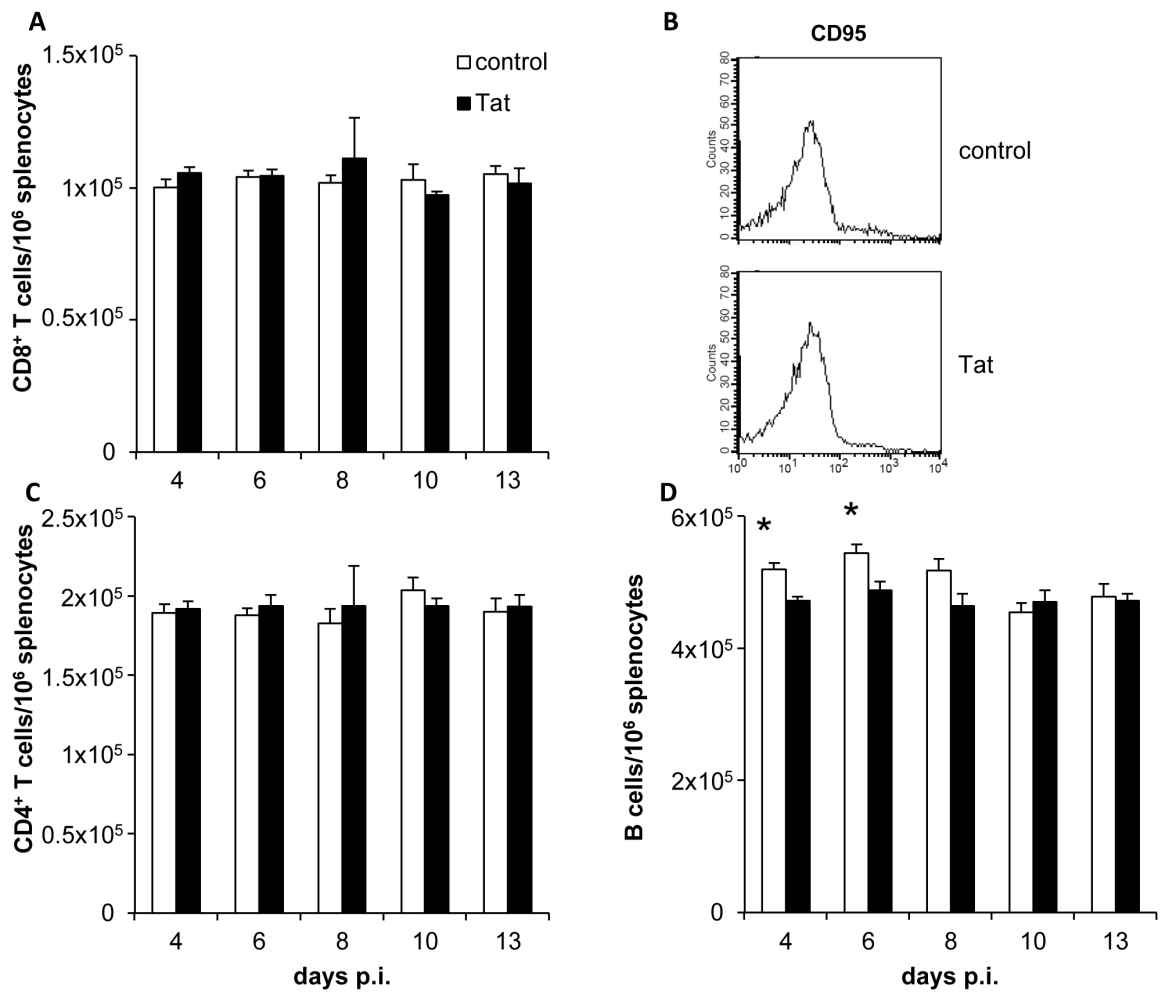
Tat does not activate bystander T cells. Control and Tat-treated HSV1-infected C57/BL6 mice were sacrificed at days 4, 6, 8, 10 and 13 post-infection. CD8^+^ (**A**), CD4^+^ (**C**) and B (**D**) lymphocytes numbers were measured by flow cytometry. Data are presented as mean ± SEM of 5 mice per group. For statistical analysis two-tailed Mann Whitney test was used. *P<0.05. One representative experiment out of three is shown. (**B**) CD95 expression was measured by flow cytometry on CD8^+^ T cells. One representative experiment out of five is shown.

To investigate whether Tat treatment affects the whole CD8^+^ T cell compartment or only HSV1-specific CD8^+^ T lymphocytes, the number of the different lymphocytes subpopulations was evaluated in spleens of control and Tat-treated HSV1-infected mice at different time points after infection. As shown in Figure 4A, the number of CD8^+^ T cells was similar in the two groups during the whole course of the experiment. To exclude pro-apoptotic effects due to the Tat treatment that may hide proliferation of CD8^+^ T cells, the expression of the pro-apoptotic Fas receptor (CD95) was measured on CD8^+^ T cells derived from control and Tat-treated mice, and no differences were detected between the two groups (Figure 4B). These data indicate that Tat favors the activation of antigen-primed CD8^+^ T cells, and exclude pro-apoptotic effects exerted by Tat on bystander CD8^+^ T cells. Some studies suggest that Tat is able to induce apoptosis in bystander B and CD4^+^ T cells [41-45]. However, conflicting results have been reported by several groups, and both proliferative and pro-apoptotic effects have been ascribed to Tat [Reviewed in 46]. Assessment of spleen composition revealed no differences at the level of CD4^+^ T cell numbers between the two groups (Figure 4C). Moreover, the CD4/CD8 ratio was not modified by Tat treatment (data not shown). Of note, when measuring the number of B lymphocytes, a mild but significant loss of B cells in Tat-treated mice was observed as compared to the control group (Figure 4D). Interestingly, the effect was transient, lasted few days after Tat injection (day 4 and 6 p.i.) and quickly disappeared. Altogether, these data demonstrate that Tat does not induce proliferation or apoptosis of bystander T lymphocytes but transiently affects B cell numbers.

### The HIV-1 Tat protein modulates the composition of the antigen-specific CD8^+^ T cell memory pool

**Figure 5:**
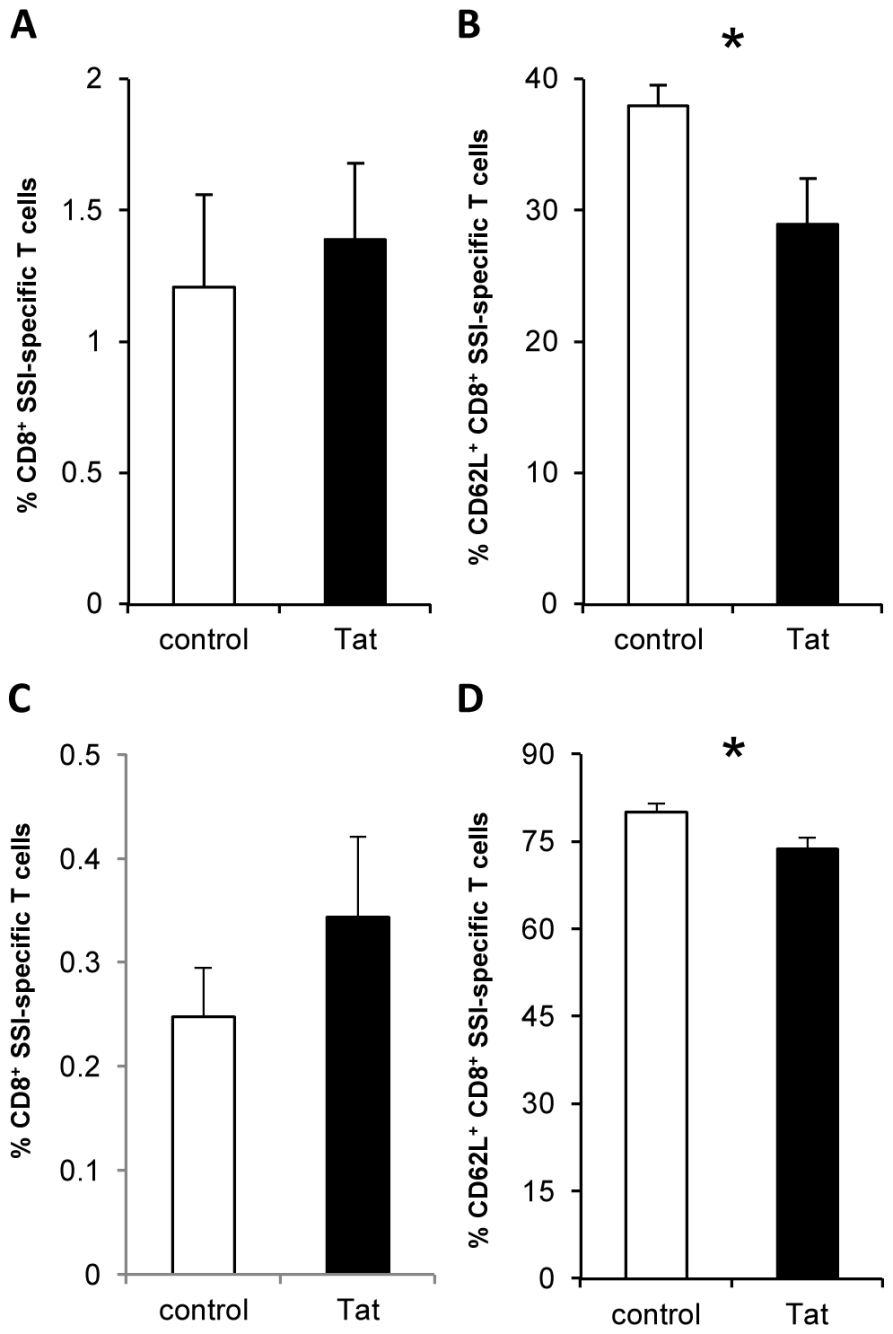
Tat administered at the time of antigen-priming favors an effector memory phenotype. Control and Tat-treated mice were infected with HSV1 wt (**A** and **B**) or with replicative-defective HSV1 (**C** and **D**) and sacrificed at days 70 post-infection. (**A** and **C**) Percentage of SSI-specific CD8^+^ T cells detected by dextramer staining. (**B** and **D**) CD62L expression was measured by flow cytometry on SSI-specific CD8^+^ T cells. Data are presented as mean ± SEM of 5 mice per group. For statistical analysis two-tailed Mann Whitney test was used. *P<0.05. One representative experiment out of three is shown.

The differentiation of memory T cells is programmed during the early phases of the immune response [47]. Since our results indicate that Tat modulates the expansion and contraction phases of antigen-specific CTL responses, we evaluated whether Tat modifies the development of the memory CD8^+^ T cell pool. To this end, HSV1-infected control and Tat-treated mice were analyzed 70 days p.i. for the presence of SSI-specific memory T cells. Data from dextramer staining (Figure 5A) and IFN-γ Elispot (not shown) showed that the numbers of epitope-specific CD8^+^ memory T cells were comparable among the two groups. The analysis of the phenotype of SSI-specific CD8^+^ T cells revealed a significant lower expression of CD62L (Figure 5B) in Tat-treated mice, indicating a larger population of HSV1-specific effector memory CD8^+^ T cells.

To exclude that the effect of Tat on T cell memory phenotype was due to HSV1 reactivation, C57BL/6 mice were infected intravaginally with a replication-defective strain of HSV (S0ZgJGFP), that primes immune responses without establishing latency, in the presence or absence of Tat. At day 70 p.i., the presence and the phenotype of SSI-specific memory T cells were determined in control and Tat-treated mice. As shown in Figure 5C, the percentage of SSI-specific CD8^+^ T cells was similar between the two groups, and SSI-specific CD8^+^ T cells from Tat-treated mice exhibited a significant lower expression of CD62L (Figure 5D), indicating that the presence of Tat during priming favors the increase of CD8^+^ effector memory T cells. Taken together, these data show that the prolonged duration and the diminished magnitude of the effector phase observed when Tat is present at the time of priming favor an effector memory phenotype. 

### The HIV-1 Tat protein induces IgG class-switching in B cells without affecting the magnitude of antigen-specific humoral responses

**Figure 6:**
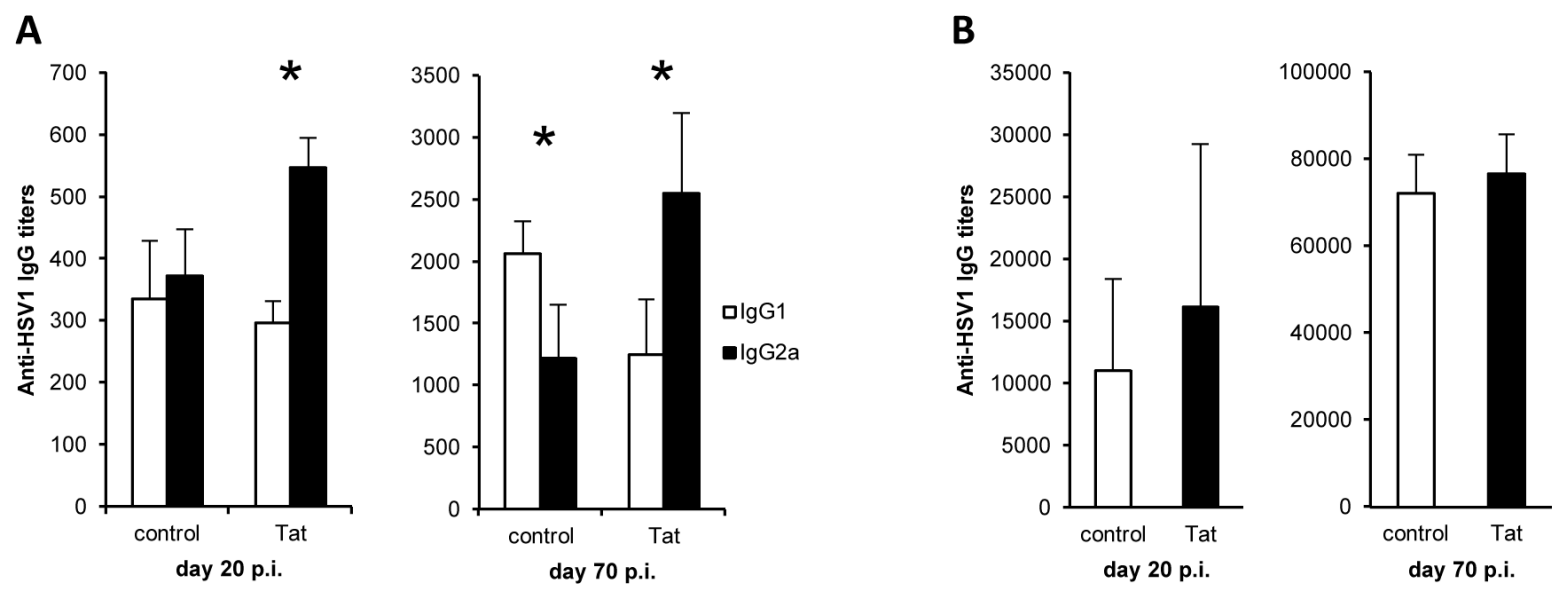
Tat administered at the time of antigen-priming favors a Th1 profile of the humoral response. Blood samples from control and Tat-treated HSV1-infected mice were collected and the presence of anti-HSV1 antibodies was detected by ELISA assay. (**A**) Anti-HSV1 IgG1 and IgG2a were measured at days 20 (left) and 70 (right) post-infection. (**B**) Total anti-HSV1 IgG were measured at days 20 (left) and 70 (right) post-infection. Data are presented as mean ± SEM of 5 mice per group. For statistical analysis two-tailed Mann Whitney test was used. *P<0.05. One representative experiment out of three is shown.

IFNγ secreted by CD8^+^ T cells is known to skew switching patterns from IgG1 to IgG2a in responding B cells [48]. Thus, as Tat prolongs IFNγ secretion, we analyzed whether the presence of Tat at the time of priming induces changes in the Th1/Th2 IgG balance. As reported in Figure 6A, at day 20 p.i., Tat-treated animals showed a prevalent anti-HSV1 IgG2a response with titers higher than IgG1 suggesting a Th1 pattern of response, whereas the control group showed a balanced anti-HSV1 Th1/Th2 pattern (similar levels of anti-HSV1 IgG1 and IgG2a). The predominant induction of antibodies associated to a Th1 response in Tat-treated mice was still evident 70 days after the infection (Figure 6A), while control mice showed a prevalence of a Th2 pattern (higher titers of IgG1). These data further demonstrate that the presence of Tat at the time of priming induces a Th1-type response [15,29,49].

To investigate whether Tat modifies the magnitude of the antiviral humoral response, we determined anti-HSV1 antibody titers in sera from Tat-treated and control HSV1-infected mice.

Specifically, mice sera collected at 20 and 70 days p.i. were tested for the presence of anti-HSV1 specific IgM (day 20 p.i.) and IgG (day 20 and 70 p.i.). No significant differences were detected among the two groups at both time points, for IgG (Figure 6B) nor for IgM (not shown). Thus, Tat modulates the quality of the anti-viral humoral response without affecting its magnitude.

## Discussion

The HIV-1 Tat protein is fundamental for viral fitness [5-7] and contributes at different levels to disease progression [3,6,14,19]. Indeed, the presence of anti-Tat cellular and humoral immune responses correlates with the control of HIV infection, as highlighted by the very early emergence of mutant variants within CTL Tat epitopes [50,51] that favor viral escape without altering Tat functions [51,52]. In addition, anti-Tat antibodies are more frequent in the asymptomatic stage of the infection and in nonprogressors [50-52], and their induction by Tat immunization in HIV-infected patients reverses signs of immune activation and T cell dysfunctions [53]. To characterize the role of Tat in T cell hyperactivation and dysfunction, we examined the effect of Tat on CD8^+^ T cell responses and antiviral immunity in different *ex vivo* and *in vivo* models of antigenic stimulation including a CTL peptide epitope, a viral antigen and a viral infection i.e. HSV that is common in HIV infection. The presence of Tat during *ex vivo* priming of naïve CD8^+^ T cells by the SSI peptide epitope, and during *in vivo* priming by the HIV-1 Gag protein and by infection with HSV1, favored the activation of antigen-specific CTLs (Figures 1, 2), but no effect was detected on bystander resting T cells (Figure 4). Effector CD8^+^ T cells generated *in vivo* in the presence of Tat underwent an enhanced and prolonged expansion that turned to a partial dysfunctionality at the peak of the response (Figure 2), that worsened acute infection (Figure 3). Thus, despite a similar phenotype during the expansion phase, CD8^+^ T cells from mice infected in the presence of Tat showed a prolonged activation as detected by cytokine release, that favored the development of effector memory CD8^+^ T cells (Figure 5) and a Th1 pattern of humoral response (Figure 6).

The Tat-mediated enhanced expansion of CTLs (Figures 1, 2A) may be due to several mechanisms. It has been demonstrated that the magnitude of the expansion of CD8^+^ T cells is related to events occurring in the first 24 hours after antigenic stimulation [54,55], and expansion requires TCR engagement (signal 1), co-stimulation (signal 2) and a pro-inflammatory environment (signal 3) [23]. Tat is known to affect all these signalling pathways. Indeed, Tat modulates antigen processing and improves the expression of certain epitope/MHC-I complexes on the surface of APCs leading to an increased stimulation of epitope-specific CD8^+^ T cells (Signal 1) [15,16,20,29,56]. Moreover, it has been demonstrated that Tat activates NF-κB [19,57,58], a transcription factor critical for the stimulation of T lymphocytes [59]. In addition, Tat modulates signal 2 enhancing CD28-mediated stimulation [6], CD40 expression [15,16], IL-2 production [6,17,19] and DC maturation and activation [15,16]. Finally, Tat is known to increase secretion of IL-12 by DCs [15,16], IFNα by macrophages [60] and of other pro-inflammatory cytokines [22,61] contributing to the type 3 signal. 

The results also demonstrate that antigen-specific CTLs primed in the presence of Tat began secreting IFNγ at earlier time after HSV1 infection (Figure 2C). However, at the peak of the response, while dextramer staining indicated the presence of more effector cells in Tat-treated mice (Figure 2A), IFNγ Elispot analysis revealed the presence of a lower fraction of functional antigen-specific CD8^+^ T cells (Figure 2B, D). Nevertheless, epitope-specific T cells were not completely exhausted, as demonstrated by the lack of PD1 up-regulation (not shown) and their recovery during the contraction and memory phases (Figures 2E, 5A). This dysfunctional status, defined as “stunning” [62], has been described in the presence of excessive stimulation [33,62-64] and coincided with a transient lower control of infection (Figure 3A). The presence of a high percentage of antigen-specific T cells not secreting IFNγ has been described in several infections [32,33,65-71]. In the case of HIV, the majority of reports show that the IFNγ secreting CTLs are about the 10-30% of tetramers-specific CD8^+^ T cells [66,69-71]. The different sensitivity of the techniques cannot explain completely the discordance between IFNγ secretion and tetramers analysis, that has been described as an impaired functionality of CD8^+^ T cells during HIV infection [2,35,67,72]. Indeed, in HIV-positive patients the loss of IFNγ secretion can be observed also in CTLs specific for other pathogens [67,68], as immune activation leads the whole T cell compartment to dysfunction, irrespectively to antigen-specificity [3]. Our data indicated that only about 30% of epitope-specific CD8^+^ T cells secretes IFNγ at the peak of the response in Tat-treated mice, while almost all antigen-specific CTLs are fully functional in the control groups (Table 1), reflecting the impairment of functionality observed in CD8^+^ T cells from HIV-infected subjects. 

**Table 1:** Percentage of Dextramer^+^ cells detected by Elispot.

Group	Peak (%)	Contraction (%)
control	92.7 ± 13.2	50.9 ± 8.5
Tat	33.3 ± 12.5	37.1 ± 2.7

Proportion of SSI-specific cells/million splenocytes, as detected by dextramer staining, secreting IFNγ in response to SSI stimulation, as detected by Elispot, at days 8 (peak) and 13 (contraction) post-infection

Although the stunned phenotype of HSV1-specifc CTLs was still evident in Tat-treated mice at day 13 p.i. (Table 1), the kinetics of antigen specific CD8^+^ T cell responses (Figure 2A) revealed that Tat-treated mice exhibited a delayed contraction of IFNγ-secreting cells as compared to the control group. It is thus plausible that IFNγ-secreting cells developed in the presence of Tat are less susceptible to death during the contraction phase. An increased survival of effectors T cells during the contraction phase has been attributed to the interaction among CD80 or CD86 expressed on DCs with CD28 expressed on the T cells [73], to Bcl-2 expression [55] and to IL-2 co-stimulation [73,74]. As Tat is known to induce DC activation and expression of CD80 and CD86 [15,16], to directly enhance CD28 co-stimulation [6], to up-regulate Bcl-2 expression [18,75] and to promote IL-2 secretion [6,17,76,77], it is tempting to speculate that these different Tat-mediated mechanisms are involved in the delayed contraction phase occurring in Tat-treated mice. Moreover, as IL-2 favors the generation of CD62L^low^ effector memory cells [78], Tat-mediated IL-2 secretion can account for the accumulation of effector memory CD8^+^ T cells observed in Tat-treated mice (Figure 5). Thus, the study of the kinetics of CTL responses in Tat-treated mice reveals that Tat prolongs the activation of CD8^+^ T cells, and this further supports a role of Tat in immune activation that turns to be deleterious for HIV-infected individuals. Indeed, despite the longer expansion phase and the delayed contraction, Tat-treated mice were unable to control the acute HSV1 infection better than control mice. Moreover, the Tat–mediated hyperactivation of CTLs promoted the accumulation of effector memory CD8^+^ T cells, a phenotype found at high frequencies in HIV-infected individuals [79-81], and reverted in HAART-treated patients by immunization with Tat [53].

Finally, we observed, for the first time in an *in vivo* model, a Tat-mediated transient loss of B cells. A role of Tat in B cell apoptosis has already been proposed [44,45,82], and B cell loss is present in HIV-infected subjects even under a suppressive HAART [3,83], and it is reverted by induction of anti-Tat antibodies in Tat-immunized subjects [53]. This suggests that Tat may, directly or indirectly, contribute to the death of B lymphocytes in HIV-positive patients. Characterization of HSV1-specific humoral responses revealed also a Tat-mediated modulation of Th1/Th2 antibody pattern in absence of any effect on the magnitude of IgG and IgM responses (Figure 6), maybe due to the prolonged IFNγ release observed in Tat-treated mice [48]. These observations further confirm previous studies demonstrating that Tat induces a predominant Th1-type adaptive immune response [15,29,49].

In conclusion, the results of this study indicate that Tat modulates CD8^+^ T cells activation and functionality resulting in a CTL response that starts earlier and lasts longer, but with a lower intensity at its peak. We propose a model by which a Tat-mediated enhancement of CTL activation and proliferation turns to be loss of functionality and accumulation of effector memory CD8^+^ T cells. The Tat protein of HIV is known to exit infected cells [9,10,12] and exert immunomodulatory effects in both non-infected and non HIV-specific T cells [14-16,18,20-22,29,77]. It is interesting to note that several Tat-mediated effects like the activation of DCs [15,16], CD4^+^ [6,19] and CD8^+^ T cells, the induction of an effector memory phenotype and the loss of B cells, as reported here, are hallmarks of the chronic immune activation observed in HIV-infected patients [2,3,79-81,83-85]. Although further studies are needed to better characterize the molecular pathways involved, we propose a key role of Tat in T cell dysfunctions and hyperactivation which may lead to an impaired control of co-infections and thus to AIDS progression. In conclusion, our data provide new insights regarding the causes of immune activation and underscore the importance of addressing anti-Tat immunity in future preventive and therapeutic approaches aimed at HIV control and cure [53,86,87].

## Supporting Information

Figure S1 **Tat does not modulate the phenotype of antigen-specific effector CD8^+^ T cells.** At day 8 post-infection splenocytes were harvested and labeled with SSI-dextramers, anti-CD8^+^, anti-CD62L and anti-CD127 monoclonal antibodies to assess the phenotype of SSI-specific CD8^+^ T cells. One representative dot plot for every group is shown.
(TIF)

Table S1 **Peptides containing K^d^-restricted CD8 epitopes from the HIV-1 Gag protein.**
(PDF)
