# Supplementary figures and images for: The HIV-1 Tat Protein Induces the Activation of CD8+ T Cells and Affects In Vivo the Magnitude and Kinetics of Antiviral Responses
Source: PLoS One. 2013 Nov 4;8(11):e77746. doi: 10.1371/journal.pone.0077746 (PMC3817196; doi:10.1371/journal.pone.0077746)

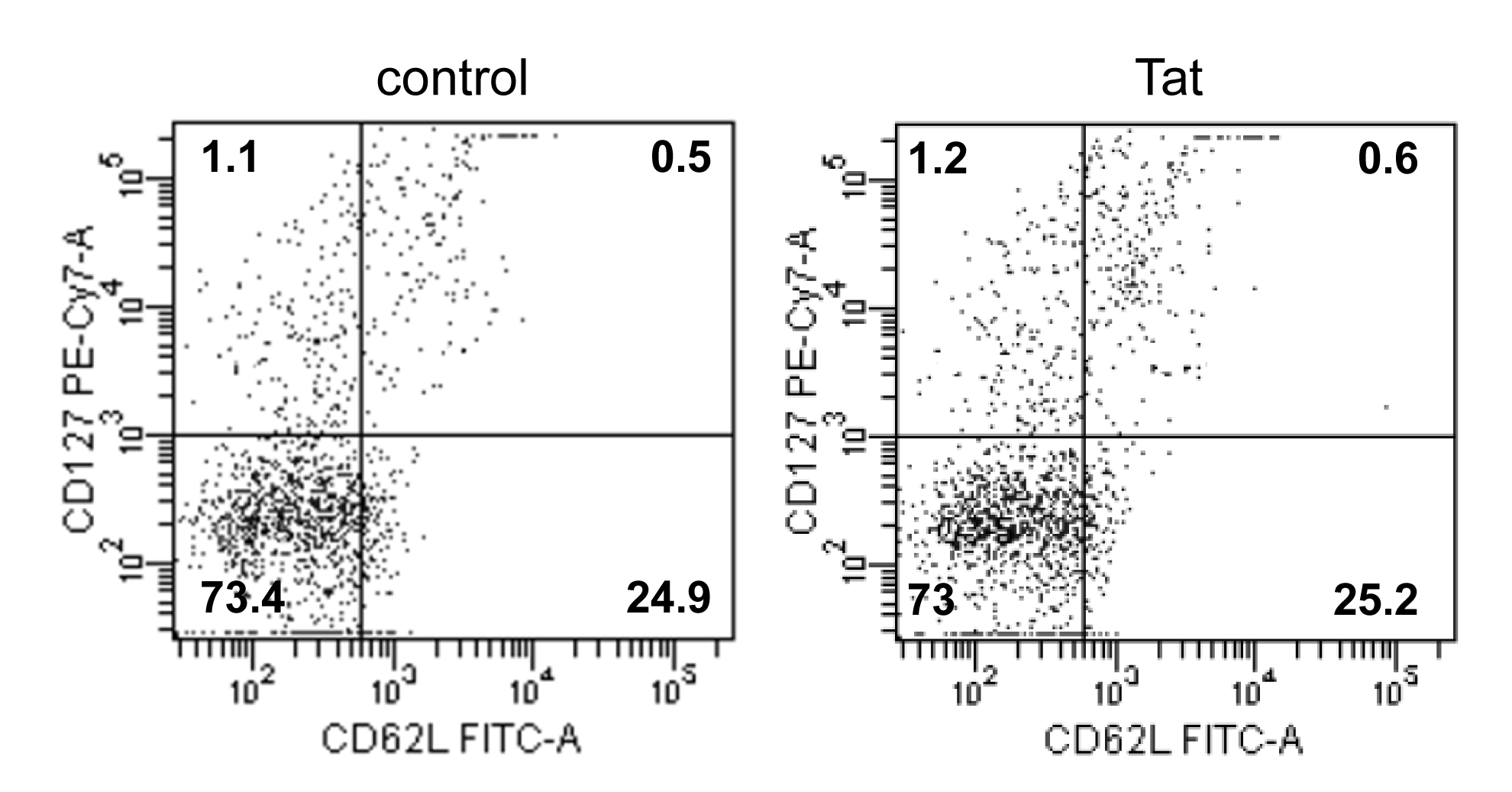

Supplement: Figure S1 — Tat does not modulate the phenotype of antigen-specific effector CD8+ T cells. At day 8 post-infection splenocytes were harvested and labeled with SSI-dextramers, anti-CD8+, anti-CD62L and anti-CD127 monoclonal antibodies to assess the phenotype of SSI-specific CD8+ T cells. One representative dot plot for every group is shown. (TIF) [file pone.0077746.s001.tif]
