# Supplementary material for: The HIV-1 Tat Protein Induces the Activation of CD8+ T Cells and Affects In Vivo the Magnitude and Kinetics of Antiviral Responses
Source: PLoS One. 2013 Nov 4;8(11):e77746. doi: 10.1371/journal.pone.0077746 (PMC3817196; doi:10.1371/journal.pone.0077746)
Supplement: Table S1 — Peptides containing Kd-restricted CD8 epitopes from the HIV-1 Gag protein. (PDF) [file pone.0077746.s002.pdf]

Epitope Sequence	Gag aa	Code
TVATLYCVHQRIEVK	Gag 81-95	TVA
SPEVIPMFSALSEGA	Gag 165-179	SPE
AMQMLKET	Gag 197-205	AMQ
AAEWDRLHPVHAGPI	Gag 209-223	AAE
IYKRWIILGL	Gag 261-270	IYK
IVRMYSPTSILDIRQ	Gag 273-287	IVR
YKTLRAEQASQEVKN	Gag 301-315	YKT
MTETLLVQNANPDCK	Gag 317-331	MTE
